# Area-Efficient Mixed-Signal Time-to-Digital Converter Integration for Time-Resolved Photon Counting

**DOI:** 10.3390/s24175763

**Published:** 2024-09-04

**Authors:** Sergio Moreno, Victor Moro, Joan Canals, Angel Diéguez

**Affiliations:** Department of Electronic and Biomedical Engineering, Faculty of Physics, University of Barcelona, 08028 Barcelona, Spain; vmoro@ub.edu (V.M.); canals@ub.edu (J.C.)

**Keywords:** CMOS, mixed-signal, time-to-digital converter (TDC), histogram, single-photon avalanche diode (SPAD), quantum dot (QD), time-resolved fluorescence

## Abstract

Digital histogram generation for time-resolved measurements with single-photon avalanche diode (SPAD) sensors requires the storage of many timestamp signals. This work presents a mixed-signal time-to-digital converter (TDC) that uses analog storage to achieve an area-efficient design that can be integrated in large SPAD arrays. Fabricated using a 150 nm CMOS process, the prototype occupies an area of only 18.3 µm × 36.5 µm, a notable size reduction compared to conventional designs. The experimental results demonstrated high performance, with an integral nonlinearity (INL) of 0.35/0.14 least significant bit (LSB) and a differential nonlinearity (DNL) of 0.14/−0.12 LSB. In addition, the proposed TDC can support the construction of histograms comprising up to 512 bins, making it an effective solution to accommodate a wide range of resolution requirements. Validated in a point-of-care (PoC) device for fluorescence lifetime measurements, it distinguished between lifetimes of approximately 4.1 ns, 3.6 ns and 80 ns with the Alexa Fluor (AF) 546 and 568 dyes and Quantum Dot (QD) 705, respectively. The analog storage design and area-efficient architecture offer a novel approach to integrating TDCs in SPAD-based systems, with potential applications in medical diagnostics and beyond.

## 1. Introduction

The field of biomedicine demands new devices that enable precise and rapid (down to nanoseconds or even picoseconds) measurement of molecular processes and interactions within biological samples. These attributes are crucial for techniques like fluorescence lifetime imaging (FLIM) [1,2,3,4,5,6], Raman spectroscopy [7,8] or time-resolved near-infrared spectroscopy [9,10]. Single-photon avalanche diode (SPAD) CMOS image sensors emerged some time ago as suitable technology in response to these requirements. These sensors offer extreme sensitivity (single-photon resolution) to low-light conditions, low noise levels and fast response times [11,12]. The Time-Correlated Single-Photon Counting (TCSPC) technique allows the detection and classification of photon arrival times with a high temporal resolution (~100 ps) [13].

In TCSPC, incident photons are timestamped using time-to-digital converters (TDCs). These circuits are usually implemented in the digital domain because they offer high accuracy, precision and noise immunity compared to analog solutions [14]. These implementations employ two primary methods: interpolation- and counter-based techniques. However, implementations of digital TDCs may suffer from high power consumption, complexity and large area requirements, resulting in a low fill factor when they are integrated with the sensors. Although interpolation-based TDCs can measure time intervals with higher resolution, their high complexity and power consumption prevent them from being the preferred alternative for high-speed applications [15,16]. On the other hand, a counter-based TDC with standard CMOS technology is the preferred option to simplify system integration. Based on the literature, several architectures of TDCs, such as digital counters, per-pixel TDCs, shared column TDCs or global TDCs [17,18,19], combined with single-photon avalanche diodes, have been used to perform Light Detection and Ranging (LIDAR) [20], 3-D imaging [21] or FLIM [22] measurements.

Several authors have reported the integration of SPADs and TDCs in optical sensing applications. Sui et al. [23] used a large CMOS SPAD detector of 30 µm for time-of-flight (TOF) and TCSPC applications, capable of measuring 320 ns of full-scale range with 312 ps resolution and an in-pixel 10-bit Vernier TDC. In another study, Perenzoni et al. [24] reported a SPAD of 10 µm in a pixel size of 38.5 × 33.5 µm^2^ designed in a 0.15 µm CMOS technology for single-point 3-D Direct TOF (DTOF) measurements. They showed a reconfigurable switching matrix of 5 × 5 pixels connected through 25 shared TDCs with 11-bit and 48 ps resolution. With them, a 50 ns full-scale range can be achieved. Henderson et al. [25] developed a 192 × 128-pixel SPAD image sensor on 40 nm CMOS technology with 33 ps resolution and 12-bit TDC for FLIM applications in a pixel size of 18.4 × 9.2 µm^2^. Erdogan et al. [26] used a 51.20 ps mean time resolution, configurable from 51.20 ps to 6.55 ns per bin, and a 512 × 16 SPAD-based line sensor with 0.13 µm CMOS technology for FLIM applications. It integrated a TCSPC mode (192.4 million events/s) of operation. The sensor was shown to be capable of spectrum fluorescence lifetime imaging by resolving three different fluorophore populations. These detectors with integrated TDCs, especially those utilizing the TCSPC method, require significant chip area.

Pixel electronics with analog solutions provide a practical alternative, offering compact dimensions without compromising resolution, scalability or time range. Analog storage circuits efficiently manage data and save area by injecting a fixed charge into a capacitor from a post-event detection. Panina et al. reported in [27] a 20 × 20 compact counter array fabricated in a standard 0.35 µm CMOS technology for photon-counting applications. Each pixel contained an in-pixel analog counting circuit with tunable resolutions of 7 and 8 bits, occupying an area of 230 µm^2^. A similar strategy was followed by Diéguez et al., in [28], whose pixel design was implemented in a 0.18 μm HV-CMOS process, integrating a linear array of 5 × 1 integrated an on-chip histogram of 9 bins per pixel. Each analog pixel achieved a resolution of up to 13 bits with a pixel size of 150 µm × 50 µm. Pancheri et al. presented in [29] a SPAD image sensor that utilized analog counting pixels for time-resolved fluorescence detection. The sensor achieved high sensitivity and nanosecond timing resolution while maintaining a compact design of 25 μm × 25 μm. The sensor array had a 32 × 32 pixel distribution and was implemented in 0.35 μm CMOS technology. To generate gating signals, a digital phase-locked loop (PLL) was used.

This work presents a TDC design in a standard 150 nm CMOS process, which achieves a wide measurement time range with high resolution. The design presents a compact area by using a mixed-signal architecture that exploits the advantages of analog and digital techniques. We validated the chip operation, showing that it is possible to perform high-resolution reconstruction of time–decay curves for various fluorescent substances. This article is structured as follows: Section 2 describes the transistor-level architecture and the operation of the TDC. In addition, the experimental characterization of the circuits is detailed in Section 2. Section 3 shows the results of the validation experiments carried out for a fluorescence analysis point of care (PoC). Finally, Section 4 and Section 5 summarize the main discussions, compare them to other relevant works and present the conclusions of this article.

## 2. Materials and Methods

### 2.1. Design Description

The TDC follows a conventional architecture of fine-grained and coarse-conversion phases. The novelty is that the coarse phase was designed in the analog domain in order to take advantage of the reduced area.

**Figure 1:**
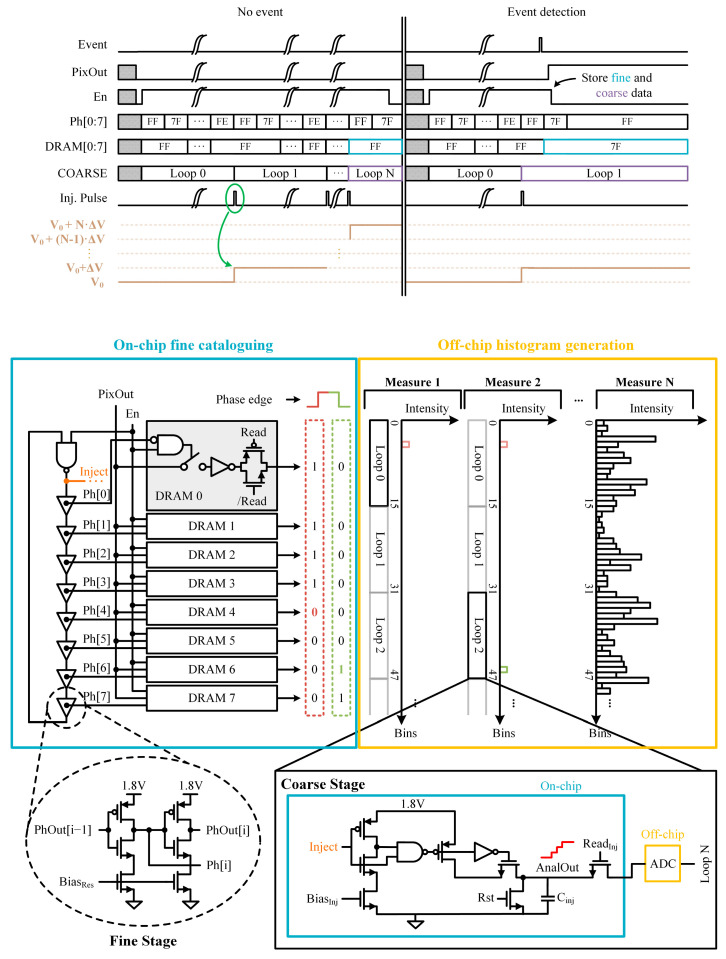
Timing diagram of the TDC full operation signals, showing no event detection and random input events during a single measuring period. Block diagram of the TDC chip, which shows the digital circuit blocks and off-chip histogram generation at the bottom. Only the main connections are indicated.

Figure 1 illustrates the block diagram of the proposed mixed-signal TDC and the operation overview. The TDC resolution is provided by the gate delay of delay cells in the fine phase (Figure 1). The fine phase is built-in, with 8 identical cells continuously rotating. We followed an approach that allowed for doubling the number of bins without adding more elements and maintaining a small area. The TDC is activated by using the rising (ascending phase) and falling (descending phase) edges of the signals. Hence, we configured the delays for the descending phases to be identical to those for the ascending phases to obtain another 8 useful bins. This resulted in 8 bins for the ascending phase and another 8 for the descending phase. After each loop of the fine stage (16 bins), the coarse phase is activated. The coarse phase (*AnalOut*) is based on an analog counter. Consequently, the temporal range is extended by taking advantage of the phases of the fine stage. The analog value is isolated to the output pad through the use of a pass transistor, which is activated by the readout signal (*Read_inj_*).

The enable signal (*En*) activates and deactivates the fine-stage operation. Detection is only allowed until the event occurs or the end of the T_exp_. The fine stage comprises eight cells of buffers based on a current starved voltage control oscillator architecture [30]. This configuration allows for low power consumption while operating at high oscillation frequencies. A standard inverting buffer drives the intermediate signal (*Ph*) to the dynamic random-access memories (DRAMs). The output signal of the buffer (*PhOut*) is connected to the input of the next delay cell. The switching time of the delay elements can be adjusted by an external bias voltage (*Bias_res_*).

The coarse phase determines the completed number of loops of the fine stage. The circuit implements an analog memory (capacitor) and an analog counter circuit based on a charge-injection scheme. The output of the NAND gate (*Inject*) activates the generation of the injection pulse. The pulse width is controlled by an external bias (Bias_inj_), which allows the step size (ΔV) in the analog memory to be increased or reduced. Each time a measurement is completed, it is necessary to read the DRAMs and the analog output signal. A metal–insulator–metal (MIM) capacitance of 200 fF is used to optimize the area covered by the TDC. This allows the injection transistors to be placed just below, occupying only 73.4 µm^2^, maintaining a simple and very compact design.

### 2.2. Experimental Characterization

**Figure 2:**
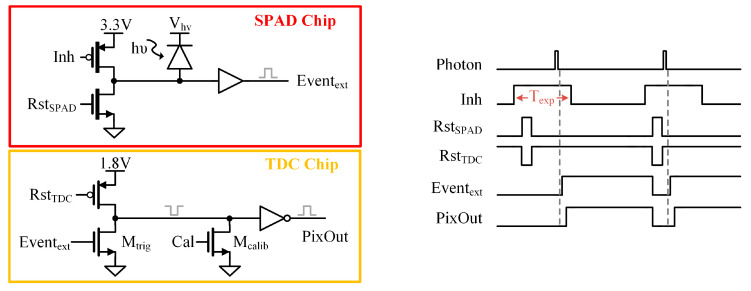
Schematics of the SPAD and TDC chip readouts. They show the interconnection between the chips and the basic operation timing diagram.

The TDC chip was characterized in an experimental setup using an external sensor-based random noise generator integrated circuit. Figure 2 presents the inhibit (*Inh*) and reset (*Rst_SPAD_* and *Rst_TDC_*) signals generated by the FPGA and used to control the sensor operation. The external sensor operation is initiated by the *Inh* signal. When *Inh* is set to a logic high state, *Rst_SPAD_* and *Rst_TDC_* are activated for a short interval to discharge the output nodes in the SPAD and TDC sides, respectively. Once reset, the circuit is ready for new detection during the exposure time fixed by T_exp_. The output signal from the external sensor (*Event_ext_*) is connected to the gate of the *M_trig_* transistor. Consequently, the final output node (*PixOut*) is at a low logic level until a new event is detected. The timing diagram of the readout control logic at different nodes is also shown in Figure 2. An additional transistor (*M_calib_*) is included in the TDC readout to calibrate the response of the fine-stage circuits without the external sensor device.

We placed the integrated circuits on a printed circuit board (PCB) to characterize the on-chip circuits. This board included an Analog-to-Digital Converter (ADC, AD7274 [31]) with a 12-bit resolution and a maximum conversion rate of 3 MS/s. In order to prevent noise in the analog signal produced when the reading switch was opened, it was necessary to read the analog value over a time period of at least 800 ns. This allowed for a sufficient settling time for the signal, thereby reducing the risk of measuring analog disturbances. Data acquisition, chip management and the generation of the calibration signals were performed with an FPGA (Cyclone IV-E on a DE0-Nano from Terasic, Hsinchu County, Taiwan [32]). External bias sources were used to generate the bias voltages *Bias_res_* and *Bias_inj_*.

The coarse stage of the TDC was characterized first. The characterization consisted of measuring the voltage step of the analog counter. The events were generated with different arrival times with the FPGA and applied to the *M_calib_* transistor, emulating the arrival of events over time. To achieve this, a delay chain was implemented utilizing the carry propagation logic [33]. This logic was designed to propagate carry signals in adders, since it has dedicated routing resources. In total, 256 carry cells were used, obtaining a total range of 10 ns approximately, with an average delay unit of 39 ps according to data extracted from the post-synthesis simulation.

**Figure 3:**
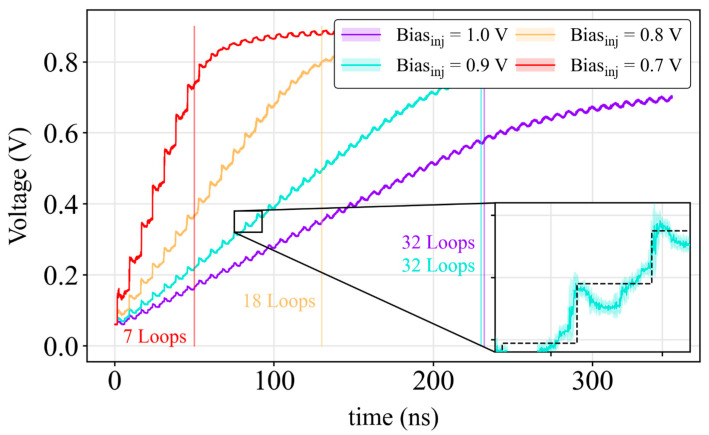
Step size of analog counter by applying different bias injection values. The dotted black line shows the ideal step size. The vertical lines indicate the resolution expressed in number of loops. Bias_res_ was 0.7 V.

Figure 3 shows the generated output voltage on the analog counter as a function of the calibration signal delay for different values of the injection bias. The TDC time range expands with the reduction in the voltage step, i.e., a decrease in *Bias_inj_*, as expected. The inset in Figure 3 shows the comparison between the ideal (dashed black line) and the measured (cyan line) step signal.

The inset of Figure 3 shows an anomalous response of the analog signal caused by pulse width variations on the different phases. Such variations are mainly consequences of the slope variations of the Inject signal. It changes slightly from phase to phase but has a pronounced effect if the phases are increasing or decreasing. This anomalous behavior limits the number of loops the circuit can complete. However, the voltage step is identified correctly while the signal remains between the reference levels:

V_0_ + (ΔV·c − 1) < V_signal_ < V_0_ + (ΔV·c) (1)

where c is the number of cycles elapsed.

**Figure 4:**
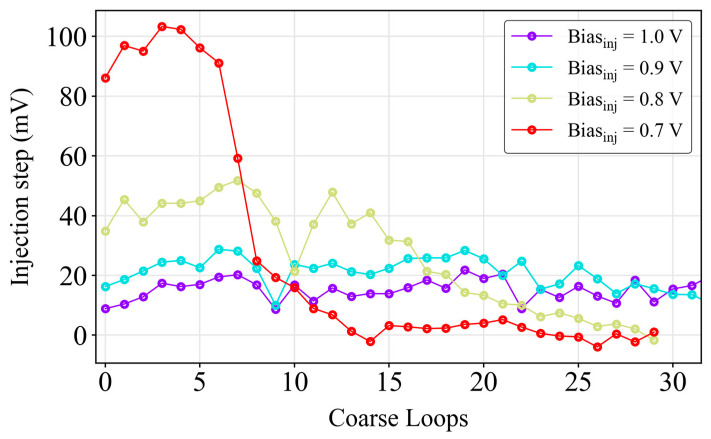
Representation of the injection step size for the purposes of the linearity study.

Figure 4 shows the injection step size as a function of the number of coarse stage loops. The time range extends from 7 loops (50 ns) for *Bias_inj_* 0.7 V (average step size: 95.8 ± 5.5 mV) up to 32 loops (230 ns) for a *Bias_inj_* of 0.9 V (average step size: 21.9 ± 3.8 mV) for a fine resolution of 450 ps (*Bias_res_* = 0.7 V). The 512-bin histogram construction configuration for the *Bias_inj_* of 0.9 V was determined to be optimal through experimentation, with a total of 32 loops (16 bins/loop) utilized. The nonlinearity of the external ADC had a negligible effect on the TDC, as it only affected the coarse stage, where the signal amplitude was significantly greater than the ADC LSB. With a 12-bit resolution, the ADC LSB (800 µV) ensured reliable TDC performance. As a result, a 5-bit analog counter was achieved, maintaining a good linearity throughout the injection steps.

**Figure 5:**
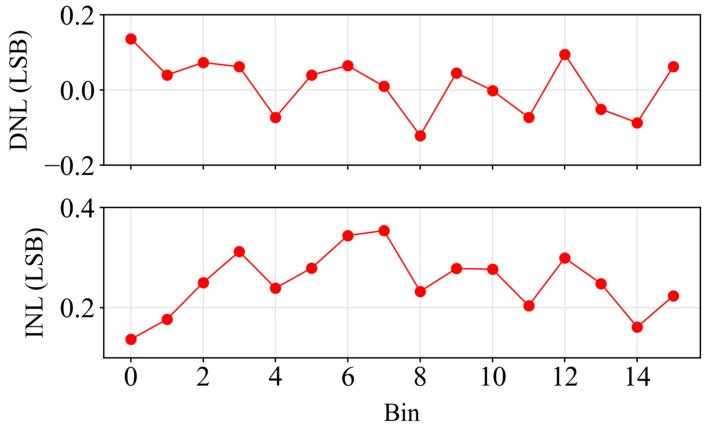
Differential nonlinearity (DNL) and integral nonlinearity (INL) of the fine stage using a bias resolution of 1.8 V.

On the other hand, the resolution of the TDC was provided by the fine stage bin width and its variation. The bin width variation was measured by performing a density test using the SPAD dark count noise [26]. Figure 5 shows the differential nonlinearity (DNL) and the integral nonlinearity (INL) obtained for the 16 bins. A similar behavior occurred for other bin widths, but only one is shown for better understanding. The DNL variation was consistently below a ±0.14 LSB, and the INL was always less than a ±0.4 LSB.

**Figure 6:**
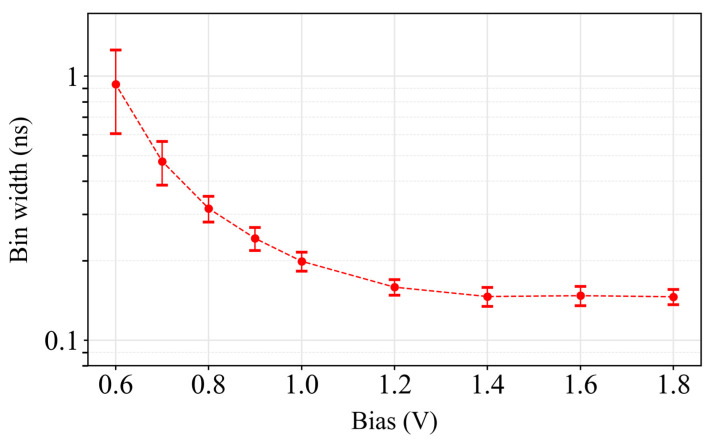
Characterization of the bin widths for several bias resolution values.

The experimental characterization of bin width as a function of bias resolution is presented in Figure 6. The mean bin size was determined as a function of the size of the 16 phases in the fine stage. The bin width could be programmed by controlling the Bias_res_ signal from 145 ps/bin up to 930 ps/bin, resulting in an overall time range from 74.3 ns to 476.5 ns, respectively. The data obtained from the DNL for each bias were used to calibrate the resulting histogram and compensate for bin-to-bin variation.

## 3. Results

### Validation Experiments for a Fluorescence-Based Point-of-Care Device

The TDC chip was validated on a fluorescence lifetime measurement setup with a SPAD detector. Fluorescence lifetime has been extensively used in several types of PoC devices [34,35]. For prior verification, the SPAD sensor and its control logic were not implemented on the TDC chip. Instead, the SPAD device used was obtained from a previously fabricated chip with a low Dark Count Rate (DCR) process, previously described in [36]. The SPAD chip consisted of a 16 × 16 SPAD array with a DCR of less than 1000 cps measured at 90% of the camera. The pixel array had a pitch of 70 μm and a fill factor of 1.6%. The photodetection probability (PDP) was approximately 10% at room temperature, with an excess bias voltage of 1.4 V. The SPAD chip incorporated active quenching based on a two-transistor configuration. It allowed an efficient and fast recovery for a new trigger operation. The SPAD was polarized at its breakdown voltage plus an overvoltage of up to 3.3 V. As explained before, the output signal of the SPAD chip was connected to the *Event_ext_* of the TDC chip.

**Figure 7:**
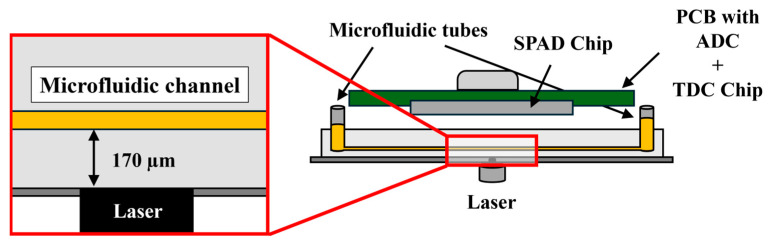
Cross-sectional view of the PoC device chamber. The laser is aligned with the camera of the SPAD chip. The fluorescence liquid is introduced into the microfluidic tubes of the µ-Slide until the microchannel is filled.

Figure 7 illustrates the cross-section of the PoC measuring chamber. This setup did not involve any complex optical system. Thus, the design was simple and low-cost for fluorescence signal detection. Simplicity and low cost are crucial for developing a PoC device for molecular diagnosis. As an excitation source, we used L405P150 [37] and PL520_B1 [38] laser diodes emitting at 405 nm and 520 nm with ~1.6 ns pulses, respectively. By changing the excitation light, it was possible to measure a large variety of organic and inorganic dyes because of the short pulse duration applied. The µ-Slide I Luer Glass Bottom from Ibidi [39] was employed as the microfluidic platform for the fluorescence measurements. A microfluidic channel with a volume of 62.5 µL and a height of 250 µm was employed to achieve uniformity in the sample preparation process for the experiments.

**Figure 8:**
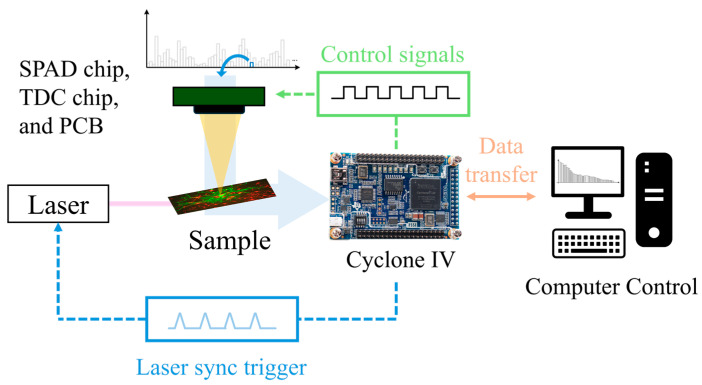
Simplified view of the measurement process flow.

Figure 8 shows an overview of the system and process flow used during the experiments. The most sensitive and lowest-noise SPAD pixel was selected to maximize the measurement accuracy. All measurements began with the excitation of the sample by activating the laser with the FPGA. The detection of the emitted light was carried out by the SPAD and synchronized with the TDC chip. There, the information regarding the arrival time of the photon was digitized in histogram format and sent to the FPGA once more. The storage and accumulation of the various arrival times were conducted in Cyclone IV. Finally, the reconstructed histogram with the lifetime was transmitted to the computer for visualization and further analysis.

With this setup, we conducted two experiments. In the initial experiment, we employed three types of fluorescence dyes: Alexa Fluor^TM^ 546 streptavidin (from Thermo Fisher Scientific, Waltham, MA, USA [40]) and Alexa Fluor^TM^ 568 streptavidin (from Thermo Fisher Scientific, Waltham, MA, USA [41]), known for their photostability and brightness in biological applications, and Quantum Dot (QD), specifically QD™ 705 ITK™ amino (PEG) (from Thermo Fisher Scientific, Waltham, MA, USA [42]), which is highly stable and resists photobleaching under extensive excitation. These conjugates, both in Phosphate Buffer Saline (PBS), emitted maximum fluorescence at 573 nm and 705 nm, respectively. The lifetimes of Alexa Fluor (AF) 546, AF 568 and QD 705 were 4.1 ns, 3.6 ns [43] and 70–100 ns [44], respectively.

**Figure 9:**
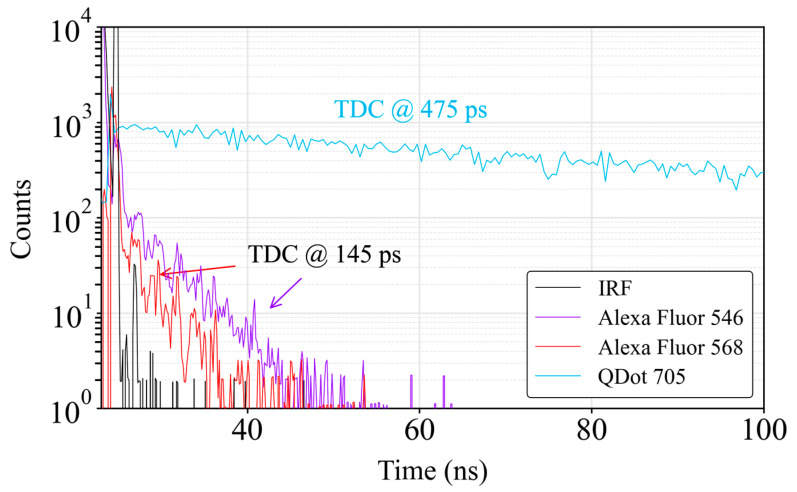
Decay times for QD 705, AF 546 and AF568 at a concentration of 1 µM. The application of the Savitzky–Golay filter in the post-processing step proved advantageous for high-resolution signals, enabling more precise measurements despite the intrinsic noise associated with the TDC.

Figure 9 shows the reconstructed histograms with the fluorescence decay measurements. The TDC resolutions were 145 ps for AF 546 and 475 ps for QD 705, respectively, which were enough to measure the lifetimes of both compounds. A total of 1 million measurements were carried out, resulting in a total exposure time of 500 ms. The decay of QD fluorophores exhibits a multi-exponential decay profile [45]. However, most of the signal contribution occurred within the first tens of nanoseconds, where the decay curve approximated a mono-exponential decay:

(2) I(t)=I0·e−tτ

where I_0_ represents the fluorescence intensity at the initial time (t = 0) while τ denotes the lifetime of the fluorophore. The lifetime of the fluorophore can be expressed as the inverse of the slope linearized in Equation (2). Taking this into consideration, linear fits were performed on the decay curves within the second region of each curve. The initial region was not eligible for consideration, as it correlated with the laser pulse. The cut-off point for the fitted data was established at three times the noise level for each concentration [37]. The resulting lifetimes for each dye were found to be 4.5 ns ± 70 ps (AF 546), 3.4 ns ± 190 ps (AF 568) and 79.7 ns ± 20 ps (QD 705), which aligns with the literature-reported values.

**Figure 10:**
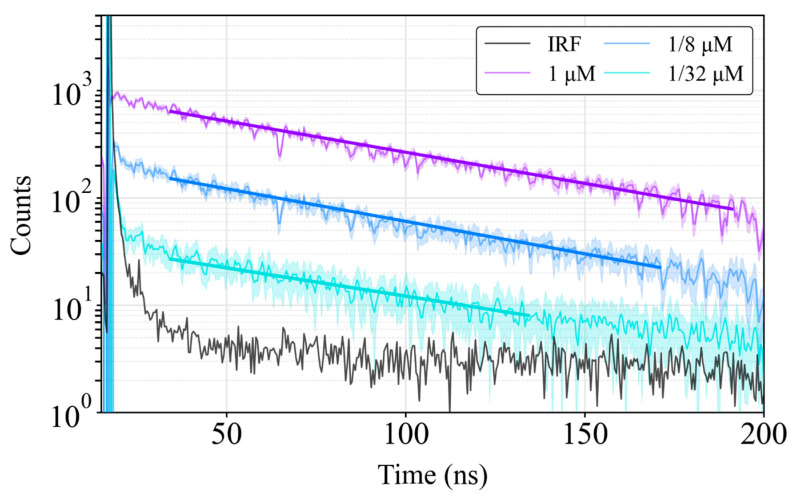
Decay histograms of the IRF and QD 705 at concentrations ranging from 1 µM to 1/32 µM. For the purpose of ease of visualization, only three concentrations are represented.

The second experiment focused on measuring the decay profiles of QDs of various concentrations. For this, the QD preparations were diluted to a concentration of 1 µM in PBS. Figure 10 presents the histograms for the fluorescence decay measurements performed on fluorophore solutions at different concentrations alongside the Instrument Response Function (IRF). The IRF determines when laser influence on decay becomes negligible. Observations indicated that beyond 40 ns, the effect of the laser tail was minimal. The average lifetime measured was 78.7 ± 0.8 ns for all concentrations, as expected.

## 4. Discussion

The principal objective of this work was to develop and demonstrate a mixed-signal TDC that employs analog storage to create an area-efficient design. The use of analog storage allowed for a notable reduction in TDC occupancy without any compromise to performance, making it an ideal solution for applications such as time-resolved measurements.

This study developed a mixed-signal TDC integrated into a circuit that occupied a total area of only 18.3 µm × 36.5 µm. For purposes of comparison, previous research is presented in Table 1. As commented before, the fine and coarse module strategy is a widely utilized approach. In references [46,47], the authors demonstrated an all-digital implementation with a compact area using 3-bit and 4-bit delay lines for the fine stage, respectively, and 7-bit and 6-bit ripple counters for the coarse stage. In [48], the coarse stage was increased to 9 bits, which resulted in an increased area. A mixed-signal TDC approach was implemented in [49] using a ring oscillator and a multi-bit interpolation scheme. The circuit was designed to capture the intermediate points within each ring oscillator cycle. The circuit was compact because it integrated custom-designed registers in the coarse stage, achieving an 8-bit TDC capable of measuring up to 50 ns with a precision of 240 ps. Our mixed-signal design demonstrated reduction of the area required by approximately 65% or more when compared to digital designs. The use of the analog coarse stage, based on an analog counter in conjunction with the fine stage (ring oscillator), facilitated the implementation of a more compact design while simultaneously offering high performance (9 bits) without an increase in the requisite area compared to [49]. The integrated circuit allowed measuring of a wide temporal range of up to 403 ns (from 74 ns to 477 ns). This feature allowed adaptation of the TDC configuration to different environmental conditions and measurement requirements, thus reducing the need for separate TDC designs. The experimental results demonstrated an INL of 0.35/0.14 LSB and a DNL of 0.14/−0.12 LSB. In addition, the TDC can support the construction of histograms with up to 512 bins, thereby demonstrating its ability to cover a wide temporal measurement range.

Digital TDCs require larger areas and are more constrained in terms of scalability when compared to mixed-signal implementation. The results indicate that mixed-signal TDC with analog storage achieves significant area efficiency, making it a viable option for integration into large SPAD arrays. The INL and DNL metrics suggest that the analog storage approach does not sacrifice accuracy or precision. The TDC configuration permits precise resolution adjustment directly on the chip. The versatility of this structure allows it to be employed in other applications where longer flight times are typical. Furthermore, in the case of lower temporal resolution, it remains less than a nanosecond, which is sufficiently precise for these applications (LiDAR, FLIM, quantum optics). The validation of the TDC on a PoC for fluorescence lifetime measurements demonstrated its capacity to differentiate between lifetimes of approximately 4 ns and 80 ns using the QD 705, AF 546 and AF 568 dyes, respectively.

Successful validation on a PoC device for fluorescence lifetime measurements underscores the practical applicability of the proposed TDC design in real-world medical diagnostics. The PoC illustrates the capability of the TDC to resolve substantial lifetime variations in fluorophores. In addition, the device was shown to be effective in resolving very close lifetimes with a difference of less than 1 ns, which aligns with recent research indicating that lifetime resolution is also required [50]. Provided that a sufficient signal is available and it remains outside the decay range of the instrument response, the SPAD sensor should perform without issues [51]. Regarding the precision of the TDC, it is important to note that the external setup is also adequate and the histogram can be processed in the same manner as described in [50]. This guarantees that the system is capable of detecting fluorophores with small lifetime differences, provided that the requisite illumination conditions are met. However, our system was limited by the PoC’s inability to excite multiple wavelengths simultaneously, highlighting the need for a new design to incorporate this capability.

## 5. Conclusions

A temporal domain circuit, fabricated in an ASIC, has been developed and demonstrated to be both compact and highly resolved. It has been validated for time-resolved fluorescence detection applications, employing a hybrid approach that integrates digital and analog circuits. The digital part consists of 8 channels and allows fine-tuning of the resolution from ~145 ps up to ~931 ps. The analog part has the potential to expand the measurement range of the circuit while maintaining a low area. Therefore, we have demonstrated a highly versatile circuit for performing histograms of up to 512 bins with an exceptional measurement time range from 74 ns up to 477 ns in a compact design of only 18.3 µm × 36.5 µm.

In our validation setup, this architecture allows for the efficient detection and characterization of a wide range of fluorescent compounds. The system was successfully employed to measure short and large fluorophores with resulting lifetimes of 3.4 ns, 4.5 ns and 78.7 ns, demonstrating its capability for a wide range of applications based on fluorescence measurements and imaging. In addition to measuring different lifetimes, the results demonstrate that the system can reconstruct fluorescence decays from very small sample concentrations (1/32 = 62.5 nM) without requiring a complex optical setup or components. Nevertheless, the current detection limit is constrained by the SPAD noise.

Consequently, the circuit is well-suited for integration into a camera that could be part of a compact, robust and simple PoC setup. Conversely, the high temporal resolution and the wide measurement range allow the exploration of other fields of application, including fluorescence microscopy based on microdisplay, LiDAR and so forth.

## Figures and Tables

**Table 1:** Performance comparison of recent TDCs.

Reference	[46]	[47]	[48]	[49]	This Work
CMOS Technology (nm)	130	130	180	150	150
Year	2011	2012	2019	2020	2024
Area (µm^2^)	2244	2000	6000	402.7	699.9 ^a^
Norm. for Process (a.u.) ^b^	132.8 k	118.3 k	185.2 k	17.9 k	31.1 k
DNL_p-p_ (LSB)	0.6	0.4	1.26	1.28	0.37
INL_p-p_ (LSB)	4	1.2	1.23	1.92	0.54
Resolution (ps)	55	119	417	210	145–931
Depth (bits)	7 (coarse) 3 (fine)	6 (coarse) 4 (fine)	9 (coarse) 3 (fine)	6 (coarse) 2 (fine)	5 (coarse) 4 (fine)
Range (ns)	55	100	-	53	74–477

^a^ The on-chip area only covered up to the digital fine stage and the analog counters, not the ADC. ^b^ The area was normalized for the process in order to facilitate a comparative analysis of implementations across different technology nodes (Area/Process^2^).

## Data Availability

The original contributions presented in this study are included in this article; further inquiries can be directed to the corresponding authors.
